# Intergenerational instructional strategies and elderly preferences for digital applications: A Malaysian case study

**DOI:** 10.1371/journal.pone.0328481

**Published:** 2025-08-14

**Authors:** Nahdatul Akma Ahmad, Muhammad Asri Mohd Ali, Tengku Shahrom Tengku Shahdan

**Affiliations:** 1 Center of Computing Sciences, Faculty of Computer and Mathematical Sciences, Universiti Teknologi MARA, Perak Branch, Tapah Campus, Perak, Malaysia; 2 School of Education and Human Sciences, Albukhary International University, Kedah, Malaysia; University of Southampton, MALAYSIA

## Abstract

**Introduction:**

As Malaysia transitions into an ageing society, older adults increasingly face challenges in acquiring digital literacy, which impacts their ability to engage with essential online services, financial transactions, healthcare applications and communication platforms. The percentage of individuals aged 65 and above in Malaysia rose from 7.2% in 2022 to 7.4% in 2023, highlighting the growing needs for digital inclusion among this demographic. While internet adoption among older individuals is increasing, many still struggle due to psychological, cognitive, and physical barriers. Factors such as low self-efficacy, fear of complexity, and age-related physical limitations hinder their effective use of digital applications. Despite government initiatives such as MyDigital and the Malaysia Digital Economy Blueprint, gaps in digital literacy persist among older adults. Research suggests that intergenerational programs (IPs), where younger individuals assist older adults in learning digital skills, can bridge this gap. These programs promote collaborative learning, reduce social isolation, and foster meaningful intergenerational relationships. However, existing instructional strategies within IPs often fail to accommodate the specific learning needs and preferences of older adults, limiting their effectiveness. Addressing these instructional gaps is essential to ensuring older adults’ successful integration into the digital world.

**Aims:**

This study aims to investigate the preferences of older adult individuals in Malaysia regarding digital applications and to propose effective intergenerational instructional strategies that enhance their digital learning experiences.

**Methods:**

This qualitative study explores older adults’ preferences for digital applications and their experiences with intergenerational instructional strategies in Malaysia. A total of 26 older adults and 13 young instructors participated in digital applications workshops at two Pusat Aktiviti Warga Emas (PAWE) centres on August, 2024. Older adults were paired with young instructors in small groups to guide them in using digital applications through hands-on learning. Data collection involved semi-structured interviews before and after the sessions, as well as observations. Thematic analysis is used to analyse interview data, identifying key insights into improving digital learning for older adults in Malaysia.

**Results:**

The study, conducted at two PAWE centres in Perak, Malaysia, explored intergenerational digital learning between 26 older adults (ages 59–82) and 13 young instructors (ages 20–24). Findings highlight the importance of structured instructional strategies, including interactive learning, direct instruction, and clear communication, to enhance digital literacy among older adults. Personalized learning approaches, small group discussions, and adapting content to individual needs improve engagement and comprehension. Smartphone familiarity plays a key role, with participants favouring WhatsApp and Facebook for communication. Older adults face challenges such as fear of complexity, physical limitations, and security concerns, while young instructors benefit from training to improve communication and instructional skills. The program fosters confidence, intergenerational bonding, and digital inclusion, demonstrating the value of tailored learning strategies in bridging the digital divide.

## Introduction

In an era characterized by rapid advancements in digital technology, the intersection of an ageing population and digital literacy has emerged as a critical area of research. The swift evolution of digital applications and services has significantly transformed various industries and improved individuals’ quality of life [1–3]. While digital technology adoption is increasing across all age groups, older adults often face challenges in keeping pace with these developments. According to the Pew Research Center [4], 88% of individuals aged 65 and above use the internet, demonstrating a growing interest in digital engagement among older adults. However, despite this increased adoption, digital literacy remains a pressing concern, particularly among ageing populations. The United Nations (UN) defines older adults as individuals aged 60 years and above, though many countries, including Malaysia, set the threshold at 65 years [5,6]. Malaysia, like many other nations, is experiencing a demographic shift toward an ageing society. According to the Department of Statistics Malaysia [7], the percentage of individuals aged 65 and above increased from 7.2% in 2022 to 7.4% in 2023. As Malaysia undergoes this demographic transformation, older adults must adapt to the digital landscape to maintain their independence and improve their quality of life [8–10]. Digital literacy, which encompasses the ability to navigate, assess, and critically engage with digital technologies, is essential for older adults. However, psychological challenges, including low self-efficacy and confidence, often hinder their engagement with digital tools [11]. Some older adults are willing to embrace new technologies, particularly through intergenerational learning programs where younger individuals guide them in digital adoption [12–15]. Nevertheless, studies have shown that despite their interest, older adults struggle to keep up with technological advancements due to cognitive and physical decline [12,16]. Addressing these challenges requires improvements in instructional strategies to enhance the effectiveness of digital learning for older adults [12].

Intergenerational programs (IP) that integrate technology provide an effective approach for fostering digital literacy among older adults. These programs encourage interaction, cooperation, and knowledge-sharing between generations [17]. Various models of IP have been implemented, such as reverse mentoring, intergenerational play workshops, and family-based learning initiatives, all of which aim to mitigate technological barriers [17–21]. However, existing instructional strategies within IPs exhibit gaps that hinder their effectiveness. Previous studies highlight issues such as inflexible learning structures, inappropriate selection of digital applications, inadequate time allocation, and suboptimal learning environments [12,15,22,23]. Addressing these gaps is crucial to enhancing digital literacy outcomes among older adults and ensuring that intergenerational instructional strategies are tailored to their needs and preferences.

## Related studies

Intergenerational programs (IPs) serve as a bridge between generations, fostering collaboration, interaction, and mutual understanding. According to Sánchez et al. [24], IPs are characterized by three key elements: [1] the involvement of participants from different generations, [2] activities designed to benefit both the individuals and society, and [3] sustained relationships based on knowledge sharing. These programs effectively reduce generational gaps, enhance digital literacy, and support older adults in overcoming technological anxieties [15,23,25]. Ahmad et al. [12] noted that older adults prefer intergenerational learning settings, particularly with younger family members, as they feel more comfortable and confident in such environments. Research on IPs highlights their benefits for both older and younger generations. Yurtseven Avci and Eren [15] observed that older adults gain a deeper understanding of younger generations by sharing life experiences and wisdom, fostering reciprocal learning and reducing age-related stereotypes [20,26]. Additionally, IPs mitigate social isolation among older adults and enhance communication skills [27,28]. The younger participants, in turn, refine their mentoring skills and technological expertise [15]. Several IP models have been implemented to promote digital literacy among older adults. Family intergenerational learning involves different generations engaging with digital tools, such as grandparents and grandchildren using interactive learning apps together [13,17,19,21]. Reverse mentoring workshops enable younger individuals to teach older adults digital skills, such as using smartphones and social media, thereby fostering confidence and competence in digital navigation [15,18,23,25,29]. Additionally, intergenerational play workshops leverage digital gaming to encourage cross-generational engagement and cognitive stimulation [14,20,30]. Mannheim et al. [26] introduced collaborative technology projects, where different age groups work together on digital initiatives, fostering teamwork and innovation.

While IPs provide numerous benefits, several challenges persist. A significant barrier to digital adoption among older adults is cognitive and physical decline. Airola et al. [31] highlighted that memory disorders hinder older adults’ ability to recall digital device usage steps, necessitating repetitive and structured instruction [13]. Furthermore, age-related physical impairments, such as reduced vision and dexterity, make certain digital interfaces inaccessible [12,30]. Psychological factors, including low motivation, technophobia, and a lack of self-efficacy, further hinder digital adoption [11,32–34]. Given these challenges, refining instructional strategies within IPs is essential to enhance digital literacy among older adults. Therefore, this study aims to investigate the preferences of older adult individuals in Malaysia regarding digital applications and to propose intergenerational instructional strategies that enhance their digital learning experiences.

## Methods

The researcher initiated contacts with the managers of two Pusat Aktiviti Warga Emas (PAWE) centres through phone calls to arrange appointments for site visits. Upon agreement, the researcher visited both centres to introduce the research objectives and propose a one-day workshop at each location. The centre managers requested an official letter and promotional materials, including a poster, to facilitate participant recruitment. Subsequently, the centres disseminated the workshop information to their members via Facebook and WhatsApp groups, while the researcher advertised the opportunity for young instructors through WhatsApp, requiring interested individuals to complete a Google Form for registration.

**Fig 1:**
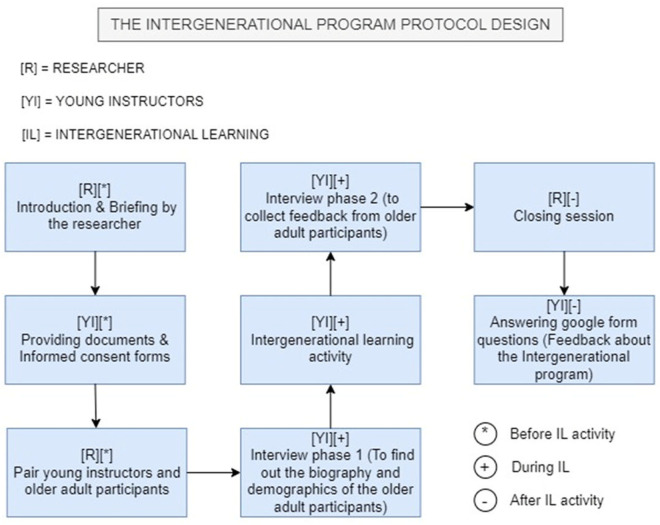
Intergenerational program flow for data collection.

The first workshop was conducted on August 26, 2024, at PAWE Tanjung Malim, and the second on August 30, 2024, at PAWE Tambun. On the day of the workshops, the researcher and young instructors arrived at the respective centres at 8:00 AM to set up the event space. Each session began with a welcoming speech by the PAWE centre manager, followed by a briefing from the researcher outlining the workshop structure and objectives for both older adult participants and young instructors. A photo session was conducted before the instructional activities commenced. The older adults were divided into small groups of 2–4 participants, where they engaged in learning digital applications with guidance from the young instructors. Various intergenerational instructional strategies, as discussed in the findings section, were employed throughout the session. The workshop concluded with a token of appreciation presented by the researcher to the centre manager and monetary tokens provided to participants as a gesture of gratitude. Both workshops followed the same procedures to ensure consistency in data collection and instructional delivery. The outline of the intergenerational program is as shown in Fig 1.

Phase 1. Introduction and Briefing

The researcher welcomed all participants including the young and older adult participants. The purpose, structure and objectives of the program were explained to ensure a clear understanding and encourage active participation.

Phase 2. Document Distribution and Informed Consent Forms

Participants received essential documents including a protocol for young instructors, semi-structured interview questions and an informed consent form. The protocol outlined the roles of young instructors in guiding older adults through digital applications. The interview questions helped facilitate discussions, which were recorded for analysis. Informed consent forms ensured that participants understood the study’s purpose, risks and benefits before agreeing to participate.

Phase 3. Pairing Young Instructors and Older Adults

Participants were divided into small groups with each young instructor paired with two or three older adults. This setup encouraged engagement and learning. Young instructors were advised to get along with the older adults before conducting interviews.

Phase 4. First Interview Session

The first interview focused on gathering older adult participants’ demographic details such as age, employment status, education level and smartphone usage. Participants had the option to skip questions they found unnecessary, such as marital status.

Phase 5. Intergenerational Learning Activity

Older adults were guided by young instructors in using digital applications. A list of 19 digital applications was provided, covering categories such as communication, multimedia, utilities and travel. Young instructors selected at least four apps they are familiar with, while older adults could choose up to two from that selection. Due to time limitations, only a limited number of apps were covered. However, older adults could also request apps which is not on the list. Young instructors demonstrated how to use the apps and encouraged hands-on practice.

Phase 6. Second Interview Session

Young instructors gathered feedback from older adults regarding their experiences. Participants shared their opinions on the program and the teaching methods utilized. The feedback was recorded for analysis later.

Phase 7. Closing Session

The researcher thanked all participants for their cooperation and collected the interview recordings from the young instructors. The program then concluded.

Phase 8. Post-Program Feedback

After the program, young instructors were contacted via WhatsApp application to complete the Google Form. The young instructors provided audio-recorded feedback on the program’s strengths and weaknesses, instructional strategies and their experiences teaching older adults. The young instructor’s insights contributed to the study’s analysis.

### Ethical approval

Ethical approval was obtained from the Universiti Teknologi MARA (UiTM) Human Research Ethics Committee (REC): Reference number: REC/01/2024(ST/MR/7).

### Inclusion criteria

Participants are older adults aged 59–85 years old. They are required to be in good health, able to manage themselves independently and have prior experience using a smartphone.

### Exclusion criteria

Participants older than 85 years old, due to possibility of health-related concerns and those who do not own a mobile phone.

### Informed consent

Participants received informed consent from the young instructor before the interviews. The instructor read and explained the details of the workshop and interview process and participants who agreed to participate completed the written informed consent form manually. Participants had the option to withdraw from the study at any point during the workshop or interview session.

### Sample size

This study employed a purposive sampling method to recruit older adults who are active members of two Pusat Aktiviti Warga Emas (PAWE) centers in Malaysia. Participants were invited to engage in a workshop conducted on August 26, 2024, at PAWE Tanjung Malim and on August 30, 2024, at PAWE Tambun. The study utilized a qualitative approach, incorporating semi-structured interviews and observations to explore older adult participants’ preferences for digital applications and their experiences with intergenerational instructional strategies. Ethical approval for this research was granted by Universiti Teknologi MARA (UiTM) Human Research Ethics Committee (REC) with reference number REC/01/2024(ST/MR/7). Before participation, all interviewees, including older adults and young instructors, were provided with consent forms to ensure informed voluntary participation.

### Findings

#### Participants demographic.

**Fig 2:**
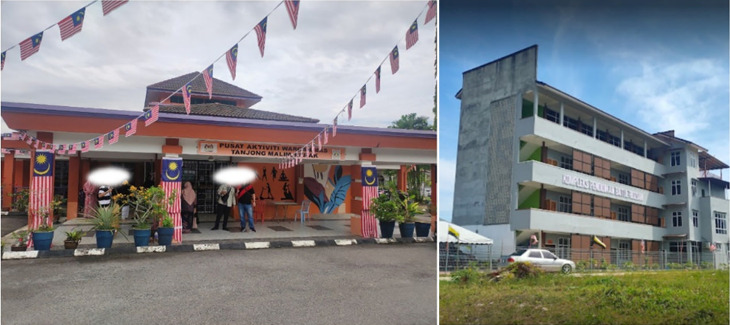
PAWE Tanjong Malim (left) and PAWE Tambun (right).

The study was conducted on-field located in Pusat Aktiviti Warga Emas (PAWE) Perak, Malaysia. There are altogether 12 PAWE centres in Perak state, including UTC Kinta, Tanjong Malim, Slim Village, Lekir, Padang Rengas, Manong, Langkap, Bagan Datuk, Tambun, Gopeng, Kampung Gajah and Taiping. Two PAWE centres among the list have been selected in this study which are PAWE Tambun and Tanjong Malim as shown in Fig 2. The selection of the case study was based on those that are active and have a larger number of participants registered with the centres. Since Perak state is expected to have most the older adult citizens by 2030 at 20.5% of the state population [35], the study concludes this location is a suitable place to host the Intergenerational Program (IP) called ‘Bengkel Pembelajaran Aplikasi Mudah Alih Antara Generasi (Warga Emas dan Belia) Tahun 2024’.

The program consisted of two groups of participants which are the young instructors and older adult individuals. The recruitment process was conducted through WhatsApp and Facebook to announce the program and registration in Google Forms for the young instructors. A total of 13 young instructors volunteered to join the program, who were almost entirely undergraduate students, except for two young instructors who were postgraduate students and unemployed. The age range of the young instructors was between 20 and 24 years old, with 7 of them having prior experience instructing older adults, usually assisting their parents or grandparents with digital applications. The young instructors recruited had experience using smartphones ranging from 3 to 15 years. Besides, 26 older adults participated and expressed interest in joining the program, consisting of ages ranging from 59 to 82 years old and were recruited using Facebook and WhatsApp. PAWE’s supervisors promoted the program by posting the event poster to provide all the necessary program information. All older adult participants were female, with most being retired, though some were still employed. Unfortunately, 2 of the 26 older adult participants did not own a smartphone and feedback during the interview session was excluded to avoid potentially misleading results. During the intergenerational program, the researcher assigned each young instructor who participated to a small group consisting of 1–3 older adult participants. Tables 1 and 2 show the demographic information of young instructors and older adult participants who participated in the program at PAWE Tanjung Malim.

**Table 1:** Demographics of older adult participants in Tanjong Malim, Perak.

Participant ID	Age	Gender	Working Status	Academic Qualification	Own Smartphone?	Young Participant ID
P1	72	Female	Unemployed	Primary School	Yes	Y1
P2	65	Female	Employed	Secondary School	Yes	Y5
P3	66	Female	Retired	Secondary School	Yes	Y2
P4	82	Female	Retired	Secondary School	No	Y5
P5	75	Female	Retired	Primary School	No	Y5
P6	69	Female	Retired	Secondary School	Yes	Y3
P7	63	Female	Employed	Certificated	Yes	Y4
P8	71	Female	Retired	Secondary School	Yes	Y4
P9	62	Female	Retired	Certificated	Yes	Y4

**Table 2:** Demographics of young instructors in Tanjong Malim, Perak.

Participant Id	Age	Gender	Academic Qualification	Status	Experience Teaching Older Adults	Used Smartphone (Years)
Y1	24	Male	Degree	Unemployed	Yes	8
Y2	22	Male	Degree	Students	Yes	12
Y3	21	Male	Degree	Students	Yes	15
Y4	23	Female	Diploma	Students	No	9
Y5	24	Male	Postgraduate	Students	Yes	12

Tables 3 and 4 show the demographic information of young instructors and older adult participants who participated in the program at PAWE Tambun.

**Table 3:** Demographics of older adult participants in Tambun, Perak.

Participant ID	Age	Gender	Working Status	Academic Qualification	Own Smartphone?	Young Participant ID
P10	70	Female	Retired	Secondary School	Yes	Y6
P11	64	Female	Unemployed	Secondary School	Yes	Y6
P12	63	Female	Retired	Degree	Yes	Y11
P13	67	Female	Retired	Secondary School	Yes	Y11
P14	60	Female	Retired	Diploma	Yes	Y10
P15	66	Female	Retired	Diploma	Yes	Y8
P16	60	Female	Retired	Secondary School	Yes	Y10
P17	59	Female	Retired	Secondary School	Yes	Y9
P18	64	Female	Retired	SRP Certificated	Yes	Y7
P19	76	Female	Retired	Secondary School	Yes	Y9
P20	59	Female	Retired	Secondary School	Yes	Y7
P21	59	Female	Retired	Degree	Yes	Y9
P22	71	Female	Retired	Postgraduate	Yes	Y13
P23	65	Female	Business	Diploma	Yes	Y13
P24	60	Female	Retired	Secondary School	Yes	Y12
P25	63	Female	Unemployed	Secondary School	Yes	Y12
P26	62	Female	Business	Secondary School	Yes	Y8

**Table 4:** Demographics of young instructors in Tambun, Perak.

Participant Id	Age	Gender	Academic Qualification	Status	Experience Teaching Older Adults	Used Smartphone (Years)
Y6	24	Male	Degree	Students	Yes	7
Y7	24	Male	Diploma	Students	Yes	13
Y8	20	Female	Diploma	Students	No	9
Y9	20	Female	Diploma	Students	No	3
Y10	20	Female	Diploma	Students	Yes	9
Y11	21	Male	Degree	Students	Yes	15
Y12	22	Male	Degree	Students	Yes	12
Y13	24	Male	Postgraduate	Students	Yes	12

#### Older adult participants’ feedback on digital applications, instructors and preferred application categories.

**Fig 3:**
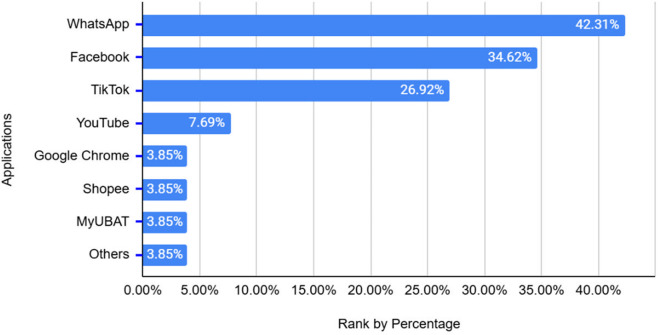
Applications are frequently used by older adults.

The bar graph in Fig 3 presents the distribution of digital applications frequently used by older adult participants. The findings indicate that WhatsApp (42.31%) is the most commonly used application among participants, followed by Facebook (34.62%) and TikTok (26.92%). These results suggest that older adults primarily engage with digital platforms for communication and social networking, which aligns with previous studies highlighting the importance of maintaining social connections through digital means [36]. In contrast, YouTube (7.69%) is used less frequently, indicating that video-based content consumption is not as prevalent among this demographic. Additionally, Google Chrome, Shopee, MyUbat and other applications (each at 3.85%) have significantly lower usage rate, suggesting that online browsing, e-commerce and healthcare-related applications are not widely adopted by older adults. These findings reflect that while older adults actively use communication and social media applications, their engagement with more complex digital tools remains limited. However, as digital literacy improves, there may be an increasing interest in exploring diverse applications beyond social interaction.

**Fig 4:**
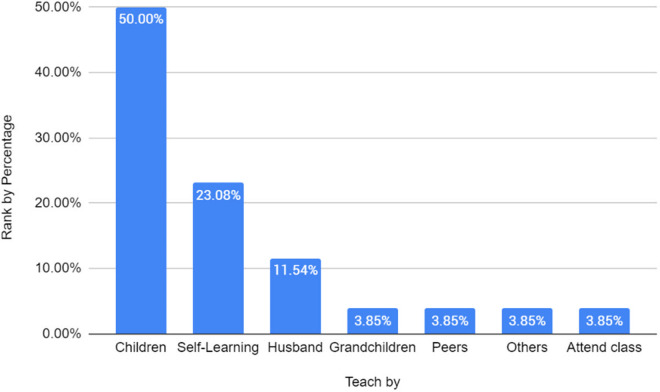
Who teaches older adults to use digital applications.

The bar graph in Fig 4 illustrates the sources from which older adult participants learned to use digital applications. The majority (50%) reported that their children served as their primary instructors, indicating that younger family members play a crucial role in introducing and guiding them in digital technology. Additionally, 23.08% of participants preferred self-learning, suggesting that a significant portion of older adults are willing to explore digital applications independently, possibly through trial and error or online resources. A smaller percentage (11.54%) relied on their husbands for instruction, which may reflect similar levels of digital literacy among spouses and a limited role in technology learning within couples. Other sources, including grandchildren, peers, other individuals and formal classes, each accounted for 3.85% of responses, highlighting that while alternative learning methods exist, they are far less common. Despite the strong reliance on children for digital instruction, many older adult participants expressed that the suitability of an instructor is not necessarily based on a close relationship but rather on the instructor’s knowledge and ability to teach effectively. However, given their limited alternatives such as spouses or peers with similar digital literacy levels, most participants acknowledged the importance of younger generations in supporting their adoption of digital technologies.

**Fig 5:**
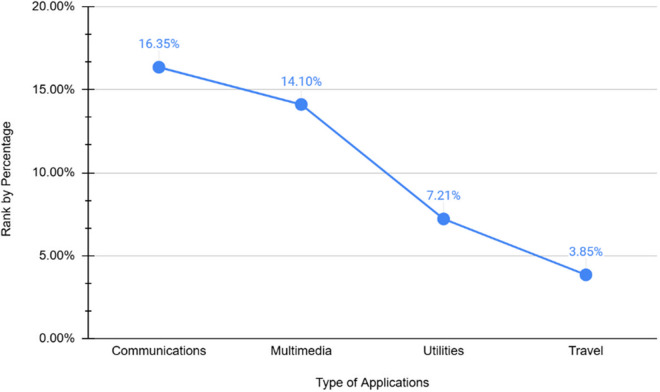
Categories of applications that have been used by older adults.

Based on the feedback from the interview sessions, the majority older adult participants reported frequent use of communication applications (16.35%) compared to other categories, as shown in Fig 5. Despite this high usage, observations indicated that many participants were no longer interested in learning about communication applications, as they were already familiar with their functionalities and interfaces. Consequently, they expressed curiosity about other applications and frequently requested demonstrations from young instructors. In some cases, participants sought to explore more complex applications, such as becoming a merchant on TikTok Shop, paying bills online, scheduling medical appointments and using online banking services. Multimedia applications (14.10%) were the second most commonly used, reflecting interest in entertainment and media consumption. However, utilities applications (7.21%) and travel applications (3.85%) had lower adoption rates, indicating limited engagement with practical and travel-related digital tools. Additionally, many older adult participants demonstrated engagement in learning digital applications by taking notes during intergenerational discussions. Upon recognizing the benefits of using a diverse range of applications, many participants expanded their curiosity and expressed interest in exploring applications beyond the provided list. Moreover, most participants identified as Muslim, which influenced their interest in religious applications such as Al-Quran recitation apps and faith-based video platforms.

#### Intergenerational instructional strategies.

Observation from the intergenerational technology-based program revealed that young instructors employed various instructional strategies to effectively engage older adult participants. Although training sessions were initially simplified before the commencement of the program, the ability of young instructors to adapt their instructional approaches ensured alignment with the learning needs of older adult participants. Based on feedback from both generations, as shown in Table 5, six key instructional strategy categories emerged, which are Direct Instruction (DI), Learner-Centered Approaches (LCA), Appropriate Pacing and Communication (APC), Interactive Learning (IL) and Reinforcement Strategies (RC). These categories represent the diverse teaching techniques implemented to enhance learning experiences within the program.

**Table 5:** Instructional strategy categories mentioned by both generations.

Feedback	DI	LCA	APC	IL	AL	RC
“For me, I want them to show me how they install it, and then teach me step-by-step on how to do everything. …” (P12, 30/8/2024, 10:30 AM)	/					
“Initially, I ask them which application they want to learn. They will install the app, and I will show them how to register as a new user. Then, I demonstrate the steps on one phone and guide the other older adult participants. Once they successfully log in, I explain each function within the app, explaining what each button does, and finally, how to use the available functions. …” (Y4, 27/8/2024, 12.30 PM)	/					
“I teach the older adults by explaining what I know and what is available in the applications. Then, I explain step by step, for example, how to deposit money, how to top up, where pressing this button will take them, and where pressing that button will lead. I also show them how to search. …” (Y8, 30/8/2024, 12:30 PM)	/					
“I teach in detail, for example, step by step - from installing the app, searching for it in the Playstore, how to install it, and then how to sign up and create an account. …” (Y9, 30/8/2024, 12:30 PM)	/					
“The way I teach the older adults is through demonstration, where I download the application on my own phone and show them the steps to follow so they can understand. …” (Y10, 30/8/2024, 12:30 PM)	/					
“I went through each function of the buttons in the apps, such as the buttons in TikTok - how to like, comment, share, and how to buy items in the shop, and so on. …” (Y13, 30/8/2024, 12:30 PM)	/					
“I think I like it this way such as tutoring, giving examples, and letting the older adults do it themselves, with discussions, especially when we have someone who enjoys talking.” (P3, 27/8/2024, 11:20 AM)		/				
“I teach them by asking them to imitate what I do in front of them. For example, if they want to open YouTube to watch a religious lecture, I will ask them to open YouTube in front of me, and I will observe the steps they need to take to watch the video they want to see.” (Y1, 27/8/2024, 12.30 PM)		/				
“The way I teach the older adults about applications is that I first download the app onto their phone, then I log in and sign up. After that, I teach them about each button within the app. …” (Y3, 27/8/2024, 12.30 PM)		/				
“… I let them try what I had just explained to ensure they understood. …” (Y13, 30/8/2024, 12:30 PM)		/				
“… and with them, you have to speak slowly, you can’t speak too fast.” (P2, 27/8/2024, 10:37 AM)			/			
“… When it comes to teaching the older adults, I simply explain briefly which buttons need to be pressed. … The appropriate technique for teaching older adults is not to complicate things. Explain briefly and only teach what is necessary, such as which buttons need to be pressed to access the required services.” (Y2, 27/8/2024, 12.30 PM)			/			
“… For example, with Grab, I show them how to order food, book a Grab car, and then how to order items from grocery stores. I also inform them about the benefits they can gain, such as getting discounts when shopping online.” (Y3, 27/8/2024, 12.30 PM)			/			
“First, I explained all the benefits of the apps listed so that they could understand the advantages of using them. … In my observation, the most suitable technique for teaching the older adults is by demonstrating the app to them, followed by an explanation of each button and the benefits of the application. When they understand the advantages, it creates a purpose and need for them to use the app.” (Y13, 30/8/2024, 12:30 PM)			/			
“In my opinion, it depends on the older adult individual, as each person has a different personality. Therefore, young participants need to be flexible in their explanations. For example, the older adult person I interviewed had a lot of experience using smartphones, so I chose a discussion technique to further explain the advantages of using digital applications.” (Y5, 27/8/2024, 12:30 PM)			/			
“… Sometimes I didn’t understand why there had to be only 16 people, but now I understand that each person needs to have two partners, so it’s easier to teach.” (P15, 30/8/2024, 10:30 AM)				/		
“I like discussions because if we just watch videos, we can learn, but we don’t focus. When it’s shown in front of a big group, we easily forget because there are too many people. Someone over there says one thing, and someone else says another. When we want to see something, for example, with a phone, you need to hold it close, right? So, if you’re sitting far away, those in the front may not even fully get it, and those at the back sometimes don’t understand either because it’s too far. But like this, it’s okay.” (P23, 30/8/2024, 10.30 AM)				/		
“I like this kind of casual chatting; it helps me remember things. If you give me a quiz, sometimes I forget. But when we have conversations like this, we can do things while remembering them. It’s normal for older people.” (P24, 30/8/2024, 10.30 AM)				/		
“We discuss, like how you do with us, show us, give examples. It means like a small discussion, like now.” (P22, 30/8/2024, 10.30 AM)				/		
“If I don’t know, I ask, because we’re senior citizens, we often forget.” (P25, 30/8/2024, 10.30 AM)				/		
“I teach the older adults through face-to-face physical interactions by showing tutorials and asking questions. … they can also ask questions when they don’t understand” (Y7, 30/8/2024, 12:30 PM)				/		
“In my opinion, the best way to teach the older adults is through face-to-face interactions, so they feel more comfortable asking questions. …” (Y10, 30/8/2024, 12:30 PM)				/		
“… I want the application to be simple, easy to digest, and easy to use, that’s all.” (P12, 30/8/2024, 10:30 AM)					/	
“… They also sometimes give us simple games like folding paper or something similar. They say we can’t play games like musical chairs because they’re afraid the older adults might fall. We can only play games that don’t pose any risk of injury, like falling. …” (P2, 27/8/2024, 10:37 AM)					/	
“… It’s like this, when young people teach the older adults, there’s always one question: ‘Auntie, do you understand what I’m saying?’ If she says yes, they continue, but if she says, ‘Can you repeat that?’, they have to go back to the beginning and explain again. Because sometimes older people, like me sometimes, we don’t always understand or know what the person is saying. So, we ask them to repeat it. That’s why I say it’s important to ask the older adults if they understand or not. …” (P2, 27/8/2024, 10:37 AM)						/
“… I show an example, then repeat the process so the older adults can do it themselves, pressing the necessary buttons. If they don’t understand, I will explain again, and then I give them the opportunity to ask questions in case there’s something they’re unsure about.” (Y8, 30/8/2024, 12:30 PM)						/
“… I repeat the steps if they are unclear about the process.” (Y10, 30/8/2024, 12:30 PM)						/
“… We can also repeat the steps in more detail.” (Y7, 30/8/2024, 12:30 PM)						/
**Feedback**	**DI**	**LCA**	**APC**	**IL**	**AL**	**RC**
**Frequency**	6	4	5	7	2	4

DI = Direct Instruction, LCA = Learner-Centred Approaches, APC = Appropriate Pacing and Communication, IL = Interactive Learning, AL = Active Learning, RC = Reinforcement Strategies, P = Older Adult Participants, Y = Young Instructors

The findings indicate that both young instructors and older adult participants acknowledge the significance of structured instructional strategies in facilitating digital literacy. Direct instruction (DI) was frequently cited as an essential method, as participants preferred guided demonstrations on application installation and usage. Learner-centred approaches (LCA) were also emphasized, with instructors adapting their teaching styles to accommodate individual learning preferences. Appropriate pacing and communication (APC) emerged as a crucial factor, as participants highlighted the need for clear, slow-paced explanations. Instructors ensured that technical jargon was minimized and information was broken down into manageable segments. Interactive Learning (IL) was another prominent strategy, where participants actively engaged in learning through practical exercises and guided discussions. Active Learning (AL) strategies further reinforced engagement by encouraging participants to experiment with applications independently. Moreover, reinforcement strategies (RC) played a vital role in enhancing retention, as instructors repeated key steps and provided additional support as needed. These strategies were particularly effective in addressing the learning preferences of older adult participants, as many expressed the need for repetitive instruction and opportunities for hands-on practice.

By referring to the frequency section in the table, data illustrate that interactive learning (IL) was the most frequently mentioned strategy, highlighting the importance of engagement and participation in digital learning for older adults. Direct instruction (DI) and appropriate pacing and communication (APC) were also widely emphasized, reinforcing the need for clear, structured and adaptable instructional methods. These findings underscore the importance of employing diverse instructional strategies to enhance the effectiveness of digital literacy programs for older adults. Table 6 shows the detailed explanation of each instructional strategy.

**Table 6:** Instructional strategy categories.

Instructional Strategy Categories	Detail
**Direct Instruction**	Step-by-step instruction: Break tasks into clear, sequential steps.
	Demonstration: Show how to perform a task before asking older adult learners to do it.
**Learner-Centred Approaches**	Encourage independence control: Encourage independence by allowing learners to take control after initial guidance.
	Assisting control: Provide support when necessary but allow learners to maintain autonomy.
**Appropriate Pacing and Communication**	Speak slowly: Adjust speech pace for clarity.
	Flexibility: Adapt teaching methods to accommodate different learning speeds and needs.
	Brief explanations: Provide concise information when introducing concepts.
	Detailed explanations: Offer more in-depth information as needed based on learner requirements.
**Interactive Learning**	Small group discussions: Foster collaboration and peer learning through interactive group activities.
	Q&A sessions: Engage learners by allowing them to ask questions and clarify understanding.
**Active Learning**	Simple activities: Engage learners with straightforward tasks that match their skill level.
**Reinforcement Strategies**	Repetition: Reiterate key concepts and tasks multiple times to help learners solidify understanding and improve retention.

#### Personalized learning for older adults.

Older adult participants demonstrated highly individualized digital learning preferences, selecting applications that catered to their specific needs. The formation of small group discussions between older adults and young instructors helped tailor the learning experience, allowing participants to focus on relevant digital applications. These small group settings enabled participants to ask specific questions and learn at their own pace, with instructors providing repeated explanations when necessary. The personalized learning approach resulted in high engagement levels from both generations, as the format allowed older adults to navigate digital tools with direct support. Additionally, since most of the older adult participants were native Malay speakers, young instructors adapted their communication strategies by using Malay and simplifying technical terms to prevent misunderstandings. This linguistic adaptation significantly improved comprehension and learning outcomes.

## Discussion

The next section will further explain each component involved in intergenerational instructional strategies, which include technology, content, older adults, and young instructors.

### Technology selection in intergenerational learning programs

The findings highlight the significance of technology selection, communication applications, and personalized learning approaches in fostering digital literacy among older adult participants in intergenerational programs. Older adult participants demonstrated a strong preference for smartphones over tablets due to familiarity and portability. While previous studies emphasize the need for larger fonts, simplified interfaces, and accessibility features [12,37], these factors alone were not decisive in device selection. Instead, familiarity with personal smartphones enhanced retention and comprehension, reducing confusion from different operating systems (OS) and graphical user interfaces (GUI). Teaching older adults to adjust font sizes and select user-friendly applications further improved their learning experience. Additionally, the portability of smartphones allowed older adults to access essential features such as communication tools, health monitoring, and navigation systems, contributing to greater independence and usability.

Communication applications, such as WhatsApp and Facebook, were the most widely used among older adults, primarily due to their role in preventing social isolation and maintaining connections with family and peers [38]. The ability to share information through text and video formats sparked further interest in digital learning. Moreover, the cost-effectiveness of internet-based communication technologies, such as Voice over Internet Protocol (VoIP) and Instant Messaging (IM), provided an added incentive for older adults to adopt these platforms. While their initial digital engagement was centred around communication applications, this familiarity encouraged them to explore additional digital tools that could enhance their daily lives.

### Content considerations in intergenerational learning programs

The findings of this study highlight key factors in the development of intergenerational technology-based programs, focusing on content, device selection, and communication applications. These elements are crucial in addressing the challenges faced by older adults in adopting digital technology, ensuring effective learning experiences, and fostering intergenerational collaboration. The content of an intergenerational technology-based program must be carefully structured to address the specific challenges older adults face, including cognitive and physical decline, technical difficulties, unfamiliarity with digital functionalities, and security concerns. Observations revealed that older adults often experience frustration when operating smartphones due to issues such as poor eyesight, accidental errors, and difficulty troubleshooting problems. Additionally, concerns over security threats, such as scams, further hinder digital adoption. To mitigate these challenges, the study suggests incorporating goal-oriented projects, simplified user interfaces, hands-on troubleshooting exercises, purposeful activities, and basic cybersecurity education.

Goal-oriented projects, such as creating a digital photo album, sending emails, or connecting with family via video calls, serve as practical learning experiences that enhance older adults’ familiarity with digital applications. Collaboration with young instructors fosters engagement and confidence. Additionally, providing step-by-step guides in simple language, along with video tutorials, supports retention and independent learning. To address technical challenges, intergenerational programs can assign younger participants as “tech buddies” to assist older adults with troubleshooting common digital issues. Role-playing scenarios further enhance problem-solving skills and digital literacy.

Purposeful activities help older adults understand the practical value of digital applications, including online banking, telehealth services, and e-commerce. Young instructors play a critical role in explaining digital functionalities using real-life analogies, which improves comprehension. However, adequate training for young instructors is necessary to ensure effective teaching strategies. Safe exploration of digital applications, guided by young instructors, can reduce anxiety associated with technology use. Basic cybersecurity education should be integrated to help older adults recognize phishing scams, create strong passwords, and identify suspicious links, thereby increasing their confidence in using digital tools safely.

To improve engagement, intergenerational programs should integrate flexibility in content selection. Allowing older adults to choose from a curated list of commonly used digital applications ensures relevance and interest. This personalized approach aligns with individual preferences and promotes sustained learning. Traditional intergenerational programs have often restricted content based on predetermined inclusion and exclusion criteria, limiting opportunities for older adults with diverse educational backgrounds. By offering a flexible selection process, programs can accommodate a wider range of participants and foster inclusive digital learning experiences.

### Older adult participants

The findings indicate that older adult participants in intergenerational technological-based programs own smartphones. This accessibility suggests that the lack of prior experience in using digital applications was relatively low among participants compared to those without smartphones. Many older adults demonstrated fundamental knowledge of smartphone usage, such as making calls, sending messages, and accessing digital applications for information. Furthermore, family influence, particularly from children who provided digital support at home, played a crucial role in fostering digital technology acceptance among older adults [9].

However, despite their acceptance of digital technology, several barriers hindered older adults from fully utilizing smartphones. One major challenge was the fear of complexity, where participants expressed reluctance to explore digital applications independently. Some participants noted, “For those who do not know, they do not need to open it,” and “I do not understand when a problem arises”. This fear of the unknown resulted in hesitation to engage with digital applications unless guided by others. Additionally, the perceived lack of relevance also contributed to their reluctance. Many older adults relied on basic smartphone functions and only explored new applications when demonstrated to them [39]. As one participant mentioned, “We do not even ask our children about what to press because we do not use it … When someone teaches us, then we understand”.

Physical and cognitive limitations further exacerbated their challenges in adopting digital applications. Issues such as declining vision and memory difficulties discouraged some participants, as highlighted in statements like, “If I wear glasses, I can see, but without them, I cannot,” and “Because we are senior citizens, we often forget.” These limitations negatively affected their confidence and motivation to engage with digital technology. Intergenerational technological-based programs can help mitigate these barriers by fostering digital technology acceptance among older adult participants. The mentoring approach within these programs provides hands-on guidance, allowing older adults to explore digital applications in a supportive environment. This structured support reduces fear and enhances their perceived relevance of digital applications. Moreover, forming small learning groups facilitates an adaptive learning pace, enabling older adults to process and retain information effectively despite cognitive or physical limitations.

Personalized learning approaches are also essential, given the varying preferences and educational backgrounds of older adults. The findings suggest that participants with higher educational backgrounds showed a greater inclination to explore advanced digital applications, whereas those with lower educational backgrounds preferred focusing on fundamental functions [40]. Additionally, previous experience with digital technology influenced learning needs, as those with limited exposure required more intensive support. In groups with diverse educational backgrounds and experiences, personalized learning ensures that young instructors can effectively tailor their instruction to meet the unique needs of each older adult participant.

### Young instructors

Young instructors play a crucial role in bridging the digital divide by guiding older adults through digital literacy programs. Older participants perceive young instructors as tech-savvy individuals who can offer clear step-by-step guidance. However, the effectiveness of young instructors depends on their ability to empathize with older adults, exhibit patience, and adapt to their learning styles. Intergenerational programs foster mutual learning, as young instructors not only impart digital knowledge but also gain wisdom and life experiences from older adults. Their presence increases older adults’ confidence in using digital applications and reduces digital exclusion. However, generational differences can pose challenges in communication and learning pace, requiring young instructors to adjust their instructional strategies accordingly. The use of complex terminology can create confusion among older adults; therefore, simplifying instructions and using relatable examples are essential strategies for improving comprehension.

Some young instructors may lack prior experience in teaching older adults, making it difficult for them to establish effective communication. Observations from the study indicate that initial conversations were sometimes challenging for young instructors unfamiliar with instructing methods. Providing pre-program training can enhance their ability to engage with older adults effectively, ensuring that instructions are conveyed clearly and respectfully. The program also benefits young instructors by improving their communication skills and fostering intergenerational bonding. The study found that young instructors demonstrated patience and respect by actively listening to older adults and responding with clear, simplified explanations. The informal conversations that emerged covered various topics, from digital application benefits to life experiences, strengthening mutual understanding and respect between generations.

### Study limitations and conclusions

#### Limitations.

This study provides valuable insights into intergenerational instructional strategies and older adults’ preferences for digital applications. However, several limitations must be acknowledged. First, the study primarily focuses on smartphone applications as the primary digital learning tool, which may not fully capture the potential benefits of other digital technologies, such as tablets, computers, or voice-assisted devices. Future research should explore the effectiveness of diverse digital platforms in enhancing older adults’ digital literacy. Second, while the study identifies key instructional strategies employed by young instructors, variations in their instructional experience and communication skills may impact the overall learning outcomes of older adult participants. Some instructors may struggle with simplifying technical content, adjusting their pacing, or effectively engaging participants. Future intergenerational programs should consider implementing standardized instructor training to ensure a more consistent and effective teaching approach. Additionally, the study does not assess the long-term retention of digital literacy skills among older adults beyond the program’s duration. While the findings suggest that structured instructional strategies improve engagement and learning, further longitudinal studies are necessary to determine whether older adults continue to use digital applications independently and sustain their digital competencies over time. Finally, cultural and socio-economic factors that may influence older adults’ learning preferences and technology adoption behaviors were not extensively explored. Future research should consider a more comprehensive analysis of how these contextual factors shape the success of intergenerational technology-based programs.

## Conclusion

This study highlights the significance of intergenerational instructional strategies in addressing the learning preferences of older adults for digital applications. The findings demonstrate that structured and adaptive teaching methods, such as direct instruction, learner-centred approaches, appropriate pacing, interactive learning, and reinforcement strategies, play a crucial role in facilitating digital literacy among older adults. The emphasis on interactive learning and personalized instruction further underscores the importance of engagement and hands-on practice in enhancing digital adoption. By ensuring that instructional strategies align with the needs and capabilities of older adults, intergenerational programs can create an inclusive and supportive learning environment that encourages digital participation.

The role of young instructors is particularly instrumental in bridging the digital divide and fostering digital inclusion. Their ability to provide step-by-step guidance, demonstrate patience, and adapt instructional strategies enhances the confidence and willingness of older adults to engage with digital technologies. Through clear and simplified explanations, young instructors help demystify digital applications, making them more accessible and less intimidating for older learners. Additionally, their enthusiasm and approachability create a positive and encouraging learning atmosphere that motivates older adults to explore digital tools.

Beyond benefiting the older adult participants, intergenerational programs also offer meaningful learning experiences for young instructors. Their involvement in these programs allows them to develop valuable skills such as effective communication, active listening, and adaptability. Engaging with older adults fosters mutual understanding and respect between generations, strengthening social bonds and promoting intergenerational cohesion. This dynamic learning exchange enriches the experiences of both groups, highlighting the reciprocal nature of intergenerational learning. Despite the benefits of these programs, young instructors may encounter several challenges when guiding older adult participants. Differences in learning paces, communication styles, and levels of digital familiarity require instructors to continuously adjust their teaching approaches. Some instructors may also lack prior instructional experience, making it difficult to effectively convey technical concepts. These challenges emphasize the need for comprehensive instructor training programs that equip young facilitators with essential teaching skills, strategies for effective communication, and techniques for adapting to diverse learning needs. By addressing these challenges, intergenerational programs can maximize their effectiveness and ensure a more seamless learning experience for older adults.

The findings of this study also emphasize the importance of integrating familiar digital applications and devices into intergenerational programs to support learning. Given the user-friendly nature of smartphones, they serve as an ideal entry point for older adults to gain confidence in technology use. By leveraging familiar communication applications as an initial learning tool, older adults can gradually build digital competency and extend their usage to more advanced applications. The structured integration of digital literacy content, including goal-oriented activities, hands-on troubleshooting, and cybersecurity education, further enhances older adults’ confidence and encourages long-term digital engagement.

Moving forward, future initiatives should continue refining intergenerational instructional strategies to enhance digital literacy among older adults. Further research is needed to examine the long-term impact of these programs on sustaining digital skills and promoting independent technology use among older learners. Additionally, cultural and socio-economic factors that influence older adults’ technology adoption behaviours should be explored to develop more tailored and effective digital literacy programs. Through continuous improvement and adaptation, intergenerational programs have the potential to serve as a sustainable model for digital empowerment, bridging the digital divide, and fostering intergenerational collaboration in an increasingly digital society. By prioritizing effective instructional strategies, comprehensive training for instructors, and the inclusion of familiar digital tools, these programs can significantly contribute to lifelong learning and digital inclusion for older adults.

## Supporting information

S1 File Interview questions. (PDF)

S2 File Research Data (This supporting data was shared through a public repository (Figshare) with DOI number: 10.6084/m9.figshare.29244866). (XLSX)

S3 File Interview Transcript Excerpt – Elderly Participant. (XLSX)
