# Supplementary material for: Intergenerational instructional strategies and elderly preferences for digital applications: A Malaysian case study
Source: PLoS One. 2025 Aug 14;20(8):e0328481. doi: 10.1371/journal.pone.0328481 (PMC12352645; doi:10.1371/journal.pone.0328481)
Supplement: S1 File — (PDF) [file pone.0328481.s001.pdf]

IDENTIFICATION AND APPROVAL

I understand that the research topic is related to exploring the experiences of elderly people toward using mobile applications for learning purposes. I have been informed that all interview sessions will be audio and video-recorded to support data collection. I understand that my participation in this study is voluntary, and I have the right to withdraw from the study at any time.

I understand that the spoken data will be recorded and transcribed to be used in various research reports. Information will be securely stored electronically. I understand that my personal details will be kept anonymous in any contributions used for research reports. The original form of data and personal information can only be viewed by the researcher and the research supervisor. If you have any questions about this research or your rights, please contact the investigator, Nahdatul Akma at telephone number 013-3627008.

Date :

Venue :

Time :

Objective 1) To observe the elderly's behaviour and reactions in the interview session.
2) Evaluating the user experience during the use of mobile applications among the elderly.

Respondent's Name: ____

SECTION A: DEMOGRAPHIC INFORMATION OF ELDERLY CITIZENS

1. When was your birth year? ______
2. Are you still working? ______________________________
3. If retired, what was your job before retirement?
4. What is your marital status? Single / Married / Widow / Widower
5. What is your highest educational level?

   ☐ Primary school (grades 1 – 6)
   ☐ Secondary school (Grades 1 – 5)
   ☐ LCE
   ☐ GCE
   ☐ Others: ________________
   ☐ Certificate
   ☐ Diploma
   ☐ Degree
   ☐ Postgraduate / Professional certificate
6. Do you have a mobile phone with an internet connection?

   ☐ Yes ☐ No
7. How long have you been using a mobile phone? ___________ years.

SECTION B: YOUTH DEMOGRAPHIC INFORMATION

1. When was your birth year? ______
2. What is your status? Students / Children / Neighbors / Grandchildren
3. What is your highest educational level?

   □ Primary school (grades 1 – 6) □ Diploma

   □ Secondary school (Grades 1 – 5) □ Degree

   □ Certificate □ Postgraduate / Professional certificate
4. Do you have experience teaching the elderly before?

   □ Yes □ No
5. What kind of application did you like to use before? Social media / Educational applications / Video games / health apps
6. How long have you been using a mobile phone/smartphone? ____________ years.

SECTION C: ELDERLY EXPERIENCES

1. Can you tell me what mobile application you have used?
2. What application do you always use?
3. How did you learn to use mobile applications while at home?
4. With whom did you learn to use mobile applications? Myself / Children / Grandchildren / Friends. And why?
5. Do you like it, if young people teach using the application? If yes/no, why...
6. What technology do you like to use? Phone / tablet / laptop
7. What method do you think is good for young people to use when teaching the elderly to use applications? Ex. Repeat, give quizzes, show video/audio, slow down, ask questions….

8. In your opinion, what features should mobile applications have so that they are easy to understand?
    a) What font style should be
    b) What colour should be
    c) Button criteria
    d) How to process navigation
    e) Icons/symbols
    f) language usage
    g) Notification

9. What mobile applications are you interested to learn?

    ☐ social media applications (eg….)
    ☐ hospital appointment reminder application
    ☐ fitness tracker application
    ☐ nutritional diet application
    ☐ Others. Please state: ____

SECTION D: YOUTH EXPERIENCE TEACHING ELDERS

1. *How did you teach him/her to use the application earlier?*
2. *What methods did you use to facilitate their understanding?*
3. *Was there any difficulty when he/she operated the application interface earlier? If yes, problem on what feature…*
4. *In your opinion, what is the appropriate method to use to teach the elderly to use mobile technology.*
5. *In your opinion, what features of this application need to be focused/improved to adapt its use to the elderly.*

Observational Comments by Researchers:
